# Developmental regulation of tau splicing is disrupted in stem cell-derived neurons from frontotemporal dementia patients with the 10 + 16 splice-site mutation in MAPT

**DOI:** 10.1093/hmg/ddv246

**Published:** 2015-07-01

**Authors:** Teresa Sposito, Elisavet Preza, Colin J. Mahoney, Núria Setó-Salvia, Natalie S. Ryan, Huw R. Morris, Charles Arber, Michael J. Devine, Henry Houlden, Thomas T. Warner, Trevor J. Bushell, Michele Zagnoni, Tilo Kunath, Frederick J. Livesey, Nick C. Fox, Martin N. Rossor, John Hardy, Selina Wray

**Affiliations:** 1 Department of Molecular Neuroscience, UCL Institute of Neurology, 1 Wakefield Street, London WC1N 1PJ, UK,; 2 Dementia Research Centre, Department of Neurodegenerative Disease, UCL Institute of Neurology, Queen Square, London WC1N 3BG, UK,; 3 Department of Clinical Neuroscience, UCL Institute of Neurology, Queen Square, London WC1N 3BG, UK,; 4 Division of Brain Sciences, Imperial College London, Hammersmith Hospital, Du Cane Road, London W12 0NN, UK,; 5 Strathclyde Institute of Pharmacy and Biomedical Sciences, University of Strathclyde, Glasgow G4 0RE, UK,; 6 Centre for Microsystems and Photonics, Electronic and Electrical Engineering, University of Strathclyde, Glasgow G1 1XW, UK,; 7 MRC Centre for Regenerative Medicine, School of Biological Sciences, University of Edinburgh, 5 Little France Drive, EdinburghEH16 4UU, UK and; 8 Gurdon Institute, Cambridge Stem Cell Institute and Department of Biochemistry, University of Cambridge, Tennis Court Road, Cambridge CB2 1QN, UK

## Abstract

The alternative splicing of the tau gene, *MAPT*, generates six protein isoforms in the adult human central nervous system (CNS). Tau splicing is developmentally regulated and dysregulated in disease. Mutations in *MAPT* that alter tau splicing cause frontotemporal dementia (FTD) with tau pathology, providing evidence for a causal link between altered tau splicing and disease. The use of induced pluripotent stem cell (iPSC)-derived neurons has revolutionized the way we model neurological disease *in vitro*. However, as most tau mutations are located within or around the alternatively spliced exon 10, it is important that iPSC–neurons splice tau appropriately in order to be used as disease models. To address this issue, we analyzed the expression and splicing of tau in iPSC-derived cortical neurons from control patients and FTD patients with the 10 + 16 intronic mutation in *MAPT*. We show that control neurons only express the fetal tau isoform (0N3R), even at extended time points of 100 days *in vitro*. Neurons from FTD patients with the 10 + 16 mutation in *MAPT* express both 0N3R and 0N4R tau isoforms, demonstrating that this mutation overrides the developmental regulation of exon 10 inclusion in our *in vitro* model. Further, at extended time points of 365 days *in vitro*, we observe a switch in tau splicing to include six tau isoforms as seen in the adult human CNS. Our results demonstrate the importance of neuronal maturity for use in *in vitro* modeling and provide a system that will be important for understanding the functional consequences of altered tau splicing.

## Introduction

Hyperphosphorylated, insoluble aggregates of the microtubule-associated protein tau are a pathological hallmark of a range of clinically diverse disorders termed the tauopathies, which include Alzheimer's disease (AD), progressive supranuclear palsy (PSP) and corticobasal degeneration (CBD) (1). The link between tau and neurodegeneration was confirmed with the discovery of mutations in the tau gene, *MAPT,* that cause frontotemporal dementia (FTD) with tau pathology (2,3). These mutations either alter the coding sequence of tau or the alternative splicing of *MAPT*. In the adult human brain, alternative splicing of *MAPT* generates six protein isoforms of tau, characterized by 0, 1 or 2 N-terminal inserts (coded by exons 2 and 3), and 3 or 4 C-terminal inserts, the additional insert coded for by exon 10 (4–6). Exon 3 is never included independently of exon 2, and therefore, six protein isoforms are generated: 0N3R, 0N4R, 1N3R, 1N4R, 2N3R and 2N4R (4,7). Tau splicing is developmentally regulated: only 0N3R tau is expressed in fetal stages, but all six isoforms are expressed in the adult central nervous system (CNS) with 3R and 4R tau being present in equal amounts in normal conditions (6). 1N tau isoforms account for 54% of total tau in the adult human brain, 0N tau isoforms account for 37% of total tau and 2N tau isoforms are the least abundant, accounting for only 9% of total tau proteins (7,8).

Proper tau splicing appears to be critical for neuronal health. A subset of FTD-linked *MAPT* mutations alter the splicing of tau exon 10, generally favoring an increase in exon 10 inclusion and increased expression of 4R tau isoforms, thereby disrupting the 3R:4R tau ratio. Two common haplotypes exist at the *MAPT* locus, H1 and H2, and H1 is associated with increased risk of the 4R tauopathies PSP and CBD. Functional dissection of *MAPT* haplotypes has shown that they can affect *MAPT* splicing at exons 2, 3 and 10 (9–12). In spite of this wealth of evidence supporting a role for altered tau splicing in disease pathogenesis, the molecular mechanisms linking tau splicing to disease remain poorly understood, in part due to the lack of *in vitro* models that recapitulate the diversity of tau isoforms seen in the adult human CNS.

The use of induced pluripotent stem cells (iPSC) differentiated into neurons has quickly become a widely chosen method to generate physiologically relevant models of neurological diseases, including dementia (13). Stem cells can be reliably differentiated into cortical glutamatergic neurons, the main tangle-bearing neurons in FTD (14). However, one potential limitation of using this approach to model tauopathy is that the neurons are at early stages of development; it remains to be seen if they recapitulate the tau splicing patterns seen in the adult human CNS. This is critical since many coding mutations in *MAPT* are located within the alternatively spliced exon 10. It is necessary that neurons generated from patients with mutations in exon 10 express 4R tau isoforms in order for the model to express the mutant protein (2).

In the present study, we characterized tau expression, splicing and phosphorylation in control cortical neurons and neurons derived from FTD patients with the 10 + 16 splice-site mutation in *MAPT*. The 10 + 16 mutation destabilizes a stem loop structure in intron 10 leading to a 2–6-fold increase of exon 10-containing mRNA in patients (15,16). We show that control neurons express mainly 0N3R tau, even at extended time points of up to 100 days *in vitro*. This has important implications for disease modeling, as mutations within exon 10 of tau would not be expressed in this *in vitro* system. In contrast, FTD neurons express both 0N3R and 0N4R tau isoforms over the same time course, demonstrating that the 10 + 16 mutation can override the developmental regulation of tau splicing. Finally, aging our control and FTD neuronal cultures to 1 year *in vitro* results in a switch from only 0N3R tau expression to expression of a diverse complement of tau isoforms. Together, our data show that the developmental regulation of tau splicing is faithfully recapitulated during *in vitro* corticogenesis, and this is disrupted by FTD-causing splice-site mutations in *MAPT*.

## Results

### iPSC and cortical neurons from patients with the 10 + 16 mutation in *MAPT*

**Figure 1.**
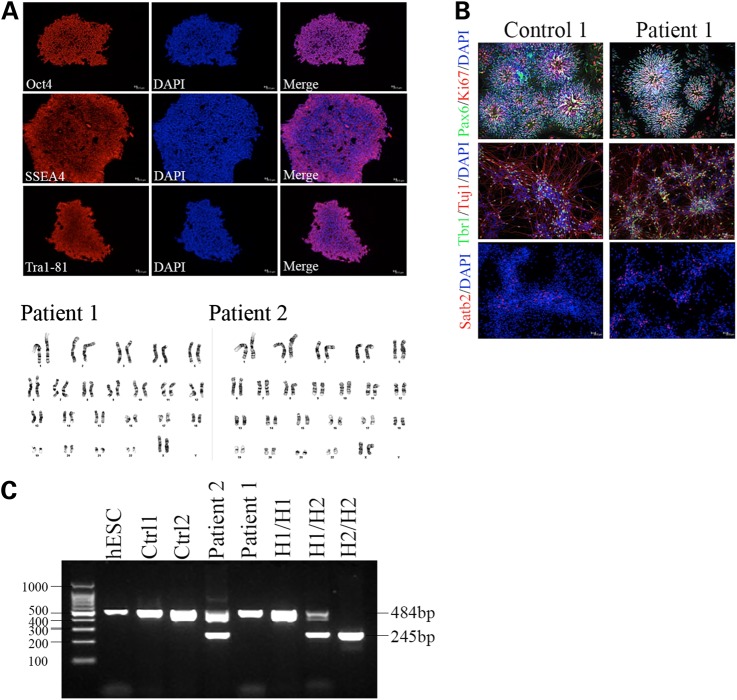
iPSC and cortical neurons from patients with the 10 + 16 splicing mutation in *MAPT*. (**A**) iPSCs were generated from fibroblasts taken from two patients with the 10 + 16 intronic mutation in *MAPT*. iPSC expressed the pluripotency markers Oct4, SSEA4 and Tra1-81 and exhibited a stable karyotype. (**B**) Control and *MAPT* iPSCs were differentiated into cortical neurons by dual SMAD inhibition followed by an extended period of *in vitro* corticogenesis. By Day 20, neural precursor rosettes were present, which were positive for the early forebrain markers Pax6 and Otx1/2. By Day 80, cells had adopted a neuronal morphology and expressed the deep-layer transcription factor Tbr1 and the upper-layer transcription factor Satb2. (**C**) The *MAPT* haplotype status of each stem cell line used in this study was analyzed using a PCR assay that detects a 238 bp deletion on the H2 background. The three control lines used in this study were homozygous for the H1 haplotype. The two FTD patients were H1/H1 and H1/H2. Human gDNAs from H1/H1, H1/H2 and H2/H2 haplotypes are included as positive controls.

Fibroblasts from two FTD patients with the 10 + 16 mutation in *MAPT* were reprogrammed into iPSC using retrovirus-mediated introduction of cMyc, Klf4, Oct4 and Sox2 as described previously (17). Resulting iPSC clones expressed the stem cell markers Oct4, Tra1-81 and SSEA4 and exhibited a normal karyotype (Fig. 1A). FTD and age-matched control iPSC were differentiated into cortical neurons by dual SMAD inhibition followed by an extended period of *in vitro* neurogenesis (14). Cortical precursor rosettes, positive for the early forebrain markers Pax6 and Otx1/2, were obtained by Day 15 of differentiation. By Day 80, neurons positive for deep-layer markers (Tbr1) and upper-layer markers (Satb2) were present in culture (Fig. 1B). Tau haplotype status of each cell line was determined using a previously described polymerase chain reaction (PCR) assay, which allows assignment of H1/H2 based in a 238 bp deletion found on the H2 background (18). All three control lines used in this study were H1/H1 homozygous (Fig. 1C). The two patients were H1/H1 homozygous (Patient 1) and H1/H2 heterozygous (Patient 2).

### iPSC-derived neurons express 0N3R tau, which is phosphorylated at multiple epitopes

**Figure 2.**
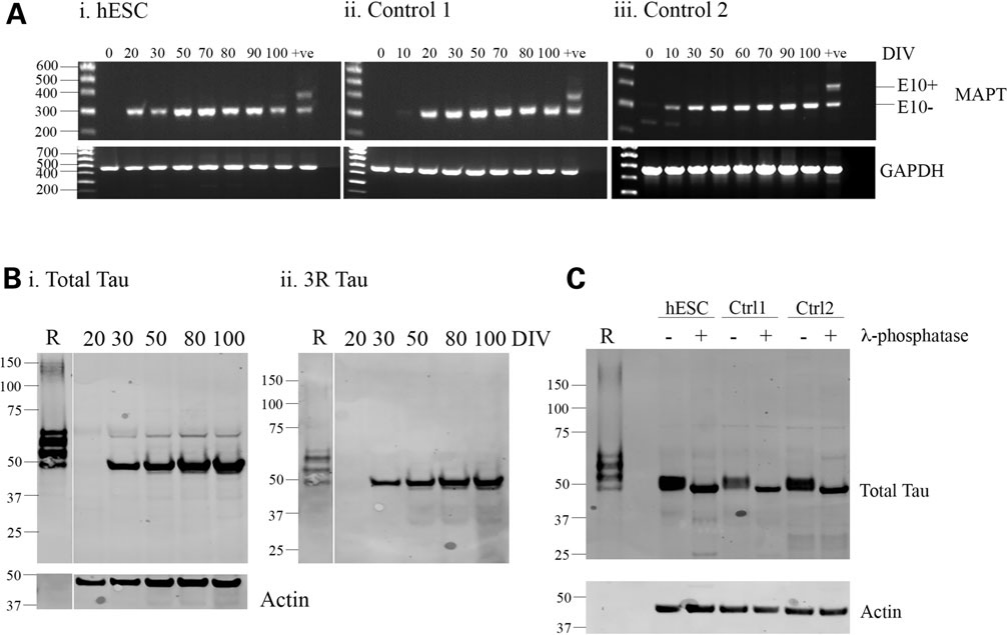
iPSC-derived cortical neurons express the fetal isoform of tau. Control iPSC and hESC were differentiated into cortical neurons, and RNA and protein were extracted at the time points indicated for analysis of tau expression and splicing. (**A**) RT-PCR with exon spanning primers located in exons 9 and 12 showed that only 3R tau was present in control neurons derived from hESC and two iPSC lines at all the time points analyzed, whereas bands of equal intensity corresponding to 3R and 4R tau were detectable in the adult human brain. (**B**) Lysates were dephosphorylated and separated by SDS-PAGE for comparison with recombinant tau ladder. Recombinant tau isoforms separate in order of decreasing molecular weight as follows: 2N4R, 2N3R, 1N4R, 1N3R, 0N4R and 0N3R. Western blots to total tau (i) showed a single band at all the time points analyzed, corresponding to 0N3R tau. This was confirmed by labeling with the 3R-specific antibody, RD3 (ii). The amount of tau present increased in a time-dependent manner. A representative image from hESC-derived neurons is shown. (**C**) Lysates from hESC and control iPSC-derived cortical neurons were collected after 100 days of differentiation and separated by SDS-PAGE alongside recombinant tau ladder. The presence of a single band after probing for total tau confirmed only 0N3R tau is present at the protein level.

As tau splicing is developmentally regulated, we set out to investigate the temporal regulation of tau expression and splicing during *in vitro* corticogenesis. Control iPSC and human embryonic stem cell (hESC) were differentiated into cortical neurons, and we examined tau expression and splicing using reverse transcriptase-polymerase chain reaction (RT-PCR) and western blot at different time points during differentiation (Fig. 2). RT-PCR using primers spanning exon 10 showed the presence of two bands of equal intensity in the human brain, corresponding to 3R and 4R tau isoforms (i.e. +/− exon 10). In contrast, although tau mRNA was readily detectable in neurons differentiated from control iPSC as early as Day 20 of differentiation, only a single band was observed following RT-PCR analysis, corresponding to 3R tau isoforms (Fig. 2A). No 4R tau mRNA was observed even at the extended time points of 100 days of differentiation.

**Figure 3.**
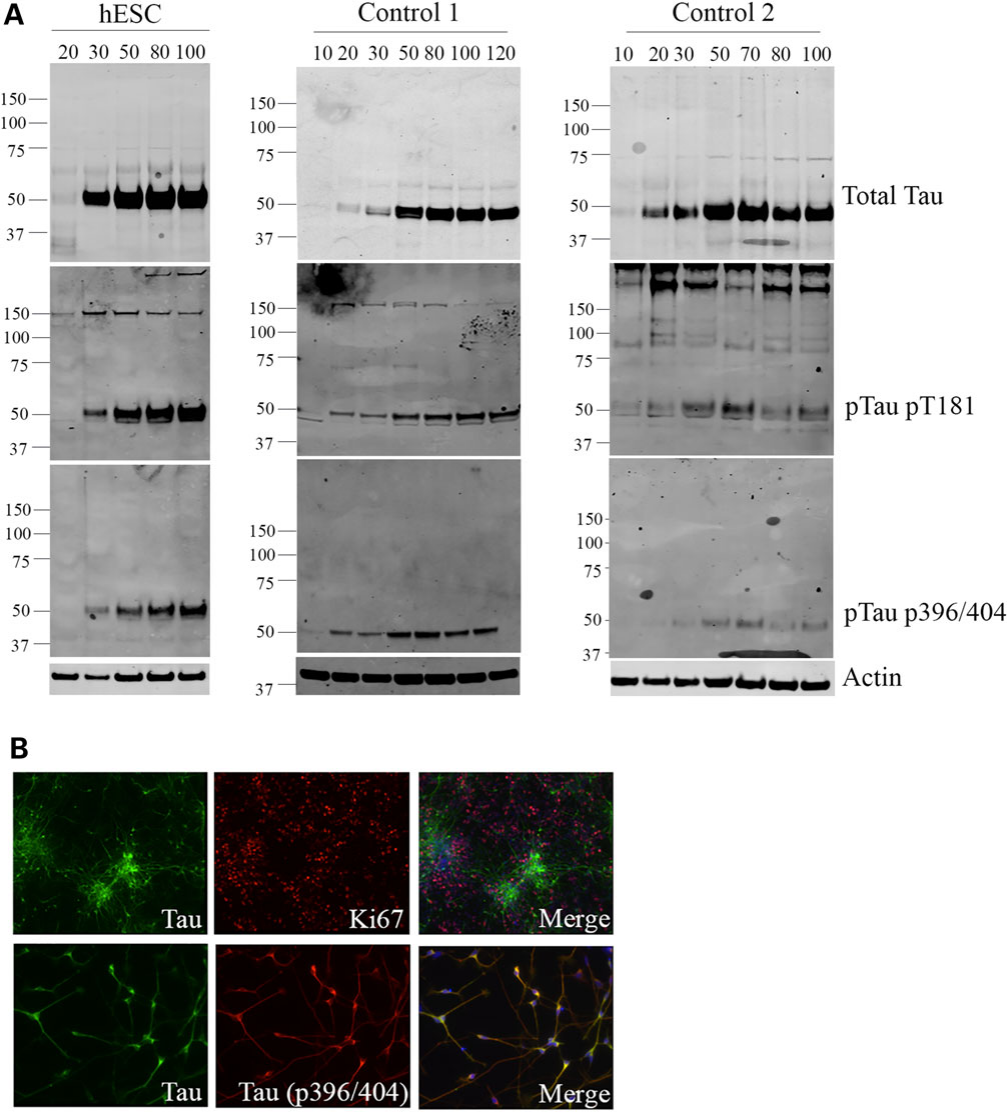
Tau is expressed in post-mitotic neurons and phosphorylated at multiple epitopes. (**A**) Whole cell lysates were collected from control cortical neurons at the time points indicated, and total and phospho tau levels were assessed by western blot. Western blots with phospho-specific tau antibodies to pT181 and pS396/S404 showed that tau is phosphorylated at multiple epitopes associated with high levels of tau phosphorylation during development. *n* = 3, independent cultures for one hESC and two iPSC lines, representative images from each line are shown. (**B**) Immunofluorescence of cortical neurons at Day 30 of differentiation showed that tau was readily detectable in neuronal cultures but did not co-localize with Ki67, a marker of dividing cells, thus indicating tau is only expressed in post-mitotic neurons. Representative images from Control 2 iPSC are shown.

This finding was confirmed at the protein level by western blot analysis (Fig. 2B). Tau protein was detectable as a single band at low concentration from Day 30 of differentiation, and the levels of tau increased dramatically between Days 20 and 30 of differentiation, which coincides with the appearance of post-mitotic neurons during differentiation (Fig. 2Bi). Dephosphorylation by lambda phosphatase followed by comparison with a recombinant tau ladder confirmed that this band corresponds to the 0N3R tau isoform, and this was also confirmed by immunoblot with the 3R-specific antibody RD3 (Fig. 2Bii). The expression of 0N3R tau was found consistently across all control lines used, as determined by analysis of dephosphorylated lysates after 100 days *in vitro* (Fig. 2C). Tau is highly phosphorylated during development, reflecting the plasticity required during neuronal development and the requirement for a dynamic microtubule network (5,19–21). Tau phosphorylation at multiple epitopes was detected in control neurons by western blot (Fig. 3A) including PHF1 (pS396/pS404) and AT270 (pT181), both of which are highly phosphorylated during development and in control brain biopsies (22).

Immunofluorescence revealed no overlap between tau and Ki67, a marker of cells undergoing mitosis (Fig. 3B), indicating that tau was expressed only in post-mitotic neurons and not in dividing neural precursors. Co-staining for total and phospho tau revealed that tau was present throughout the cell body and axon, with phospho tau mainly in axons consistent with a role for tau in microtubule remodeling during axonal outgrowth.

### Tau splicing is altered in neurons with the 10 + 16 splice-site mutation in MAPT

A large number of mutations in tau that are linked to FTD are located within exon 10. Our results with control neurons indicate that it will be difficult to model these mutations in iPSC-derived neurons, as the exon containing the mutation would be spliced out in 0N3R tau; therefore, no mutant protein would be present in the neuronal culture. We chose instead to focus on the 10 + 16 splice-site mutation, which destabilizes a stem loop in intron 10 resulting in increased production of 4R isoforms (15). FTD patients with the 10 + 16 mutation have an average age at onset of 50 years and cortical neurofibrillary tangles composed mainly of 4R tau isoforms are observed at postmortem (23–25).

**Figure 4.**
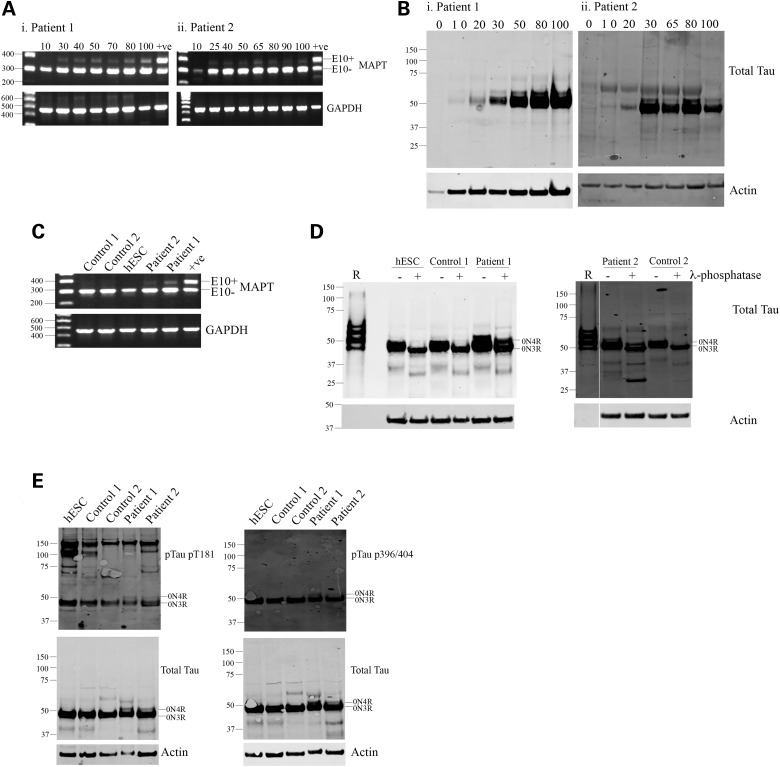
Tau splicing is altered in neurons from patients with the 10 + 16 mutation in MAPT. iPSCs from two FTD patients with the 10 + 16 intronic mutation in *MAPT* were differentiated into cortical neurons, and RNA and protein were extracted at the time points indicated for the analysis of tau expression and splicing. (**A**) RT-PCR revealed the expression of both 3R and 4R tau isoforms at all the time points analyzed. (**B**) Western blots of whole cell lysates to total tau revealed the presence of two protein bands throughout differentiation. (**C**) RT-PCR of control and 10 + 16 neurons at 100 days of differentiation confirmed a robust 4R tau expression in 10 + 16 cells and a complete absence of 4R in control neurons. (**D**) Whole cell lysates were collected after 100 days of differentiation and analyzed for tau isoform expression at the protein level by comparison with control cell lysates and recombinant tau ladder. Recombinant tau isoforms separate in order of decreasing molecular weight as follows: 2N4R, 2N3R, 1N4R, 1N3R, 0N4R and 0N3R. Dephosphorylation of protein extracts by lambda phosphatase revealed a single band in control neurons but two protein bands in FTD 10 + 16 patient cells, corresponding to the expression of 0N3R and 0N4R tau isoforms. (**E**) Phosphorylation status of tau was assessed in control and FTD neurons after 100 days of differentiation. No hyperphosphorylation of tau in FTD cells was observed; however, both 0N3R and 0N4R isoforms were phosphorylated at the epitopes examined. *n* = 3, independent cultures from each patient, representative images shown.

iPSCs from two FTD patients with the 10 + 16 mutation were differentiated into cortical neurons. RT-PCR with exon spanning primers revealed the presence of both 3R and 4R tau in neurons with the 10 + 16 mutation (Fig. 4A). This was confirmed by western blot (Fig. 4B) where two bands immunoreactive for total tau were detected. RT-PCR of control and 10 + 16 neurons at Day 100 of differentiation revealed a complete absence of 4R tau in control cells but a robust expression of exon 10 containing transcripts in FTD neurons (Fig. 4C). Dephosphorylation of whole cell lysates from control and FTD neurons after 100 days of differentiation revealed that these corresponded to 0N3R and 0N4R tau isoform expression by FTD 10 + 16 cells (Fig. 4D). This demonstrates that the *MAPT* 10 + 16 splicing mutation is able to override the developmental regulation of tau exon 10 splicing *in vitro*. Tau phosphorylation in FTD neurons was found at similar levels to control neurons (Fig. 4E), with the exception that both expressed isoforms (0N3R and 0N4R) were phosphorylated at the epitopes examined.

### Postnatal tau splicing is observed at extended time points *in vitro*

**Figure 5.**
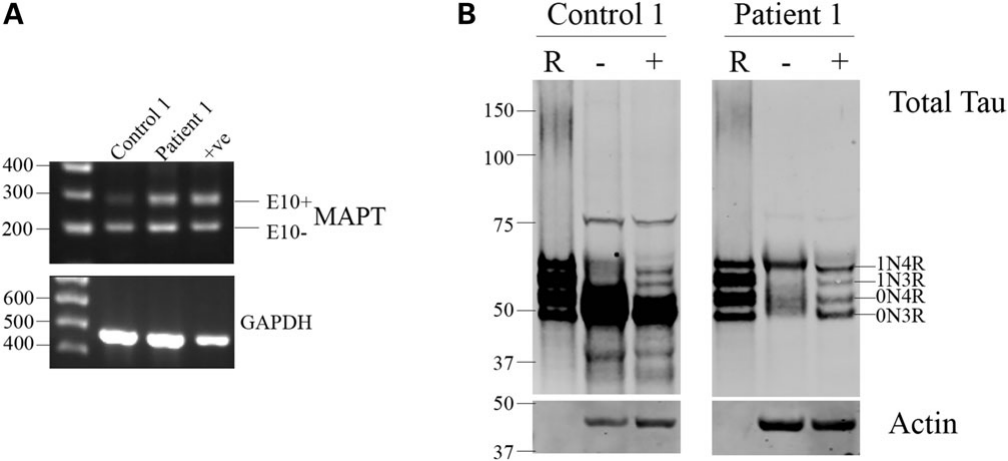
Tau splicing *in vitro* at extended time points recapitulates development. To investigate whether neurons would express a postnatal pattern of tau splicing at postnatal time points in culture, cells were aged to extended time points up to 365 days. (**A**) RT-PCR showed a robust expression of 3R and 4R tau in both control and 10 + 16 neurons after 365 days of culture. (**B**) Western blotting of lysates after dephosphorylation with lambda phosphatase revealed cortical neurons expressed multiple tau isoforms after 365 days in culture. Recombinant tau isoforms separate in order of decreasing molecular weight as follows: 2N4R, 2N3R, 1N4R, 1N3R, 0N4R and 0N3R. Control neurons expressed 0N3R, 0N4R, 1N3R and 1N4R tau. 10 + 16 neurons expressed the same complement of tau isoforms but with higher levels of 4R tau. *n* = 3, independent cultures for two control iPSC lines and one patient iPSC line.

iPSC neurons expressed only the 0N3R fetal tau isoform even after 100 days *in vitro.* We have also demonstrated that the 10 + 16 intronic mutation in *MAPT* alters tau splicing, resulting in the production of 0N4R tau and 0N3R. Although these cells provide a useful platform for studying the functional consequences of tau mis-splicing, it is desirable to have a neuronal cell model that expresses all six tau isoforms. To test the hypothesis that neurons would express postnatal patterns of tau at postnatal time points, we aged neurons in culture up to 365 days and analyzed them for tau expression and splicing. At Day 365 *in vitro*, we observed a switch in tau splicing in control neurons from only 3R isoforms to both 3R and 4R tau by RT-PCR (Fig. 5A). Dephosphorylation and alignment with a recombinant tau ladder revealed the presence of 0N3R, 0N4R, 1N3R and 1N4R tau isoforms in control cells, including both 3R and 4R variants, but still with a predominance of 0N3R (Fig. 5B). In 10 + 16 neurons, 0N3R, 0N4R, 1N3R and 1N4R were also expressed, but with an increased amount of 4R tau isoforms relative to controls. We were unable to observe 2N tau isoforms; however, these comprise only 9% of total tau in the adult brain, so it is possible that their levels are below the limit of detection in our analysis (7,8).

## Discussion

We have shown that tau splicing in iPSC-derived cortical neurons recapitulates tau expression and splicing patterns seen in human brain development. *In vitro* corticogenesis from iPSC takes 100 days *in vitro*, during this time iPSC neurons express only the 0N3R (fetal) tau isoform. This has important implications for the use of iPSC in disease modeling. It is tempting to select clinically aggressive tau mutations such as P301S for *in vitro* modeling, which can have an extremely early-onset and therefore maximize chance of observing a cellular phenotype (26). However, these mutations are located in exon 10 of the tau gene, which is spliced out in 100-day cortical neuronal cultures, and so the mutant protein would not be present. Our results are consistent with previous reports suggesting gene expression profiles of PSC-derived neurons cluster more closely with fetal rather than adult neurons (27). In contrast, expression of exon 10-containing tau isoforms has been reported in iPSC-derived dopaminergic and mixed neuronal populations between 4 and 10 weeks of differentiation (28,29). The differences observed between this and our own findings could indicate neuronal subtype-specific regulation of tau splicing. It is worth noting that in all cases, the 3R:4R ratio was still reduced compared with that observed in the adult human brain. Our results suggest that successful use of iPSC to model *MAPT* mutations will rely on the selection of mutations that are present in constitutively expressed exons.

Several reports have successfully used iPSC technology to model tau mutations; however, these have either focused on mutations located outside of the alternatively spliced exons such as the A152T variant, or have used overexpression of adult tau isoforms (30,31). A recent report described a novel tau variant, K298E, that leads to an overproduction of 4R tau when a mini gene containing this variant is transfected into neural stem cells (32). In contrast, we demonstrate for the first time in a human, neuronal, *in vitro* system that the intronic 10 + 16 splice-site mutation in *MAPT* can override the developmental regulation of endogenous *MAPT* splicing, leading to the production of 3R and 4R tau even at the earliest stages of neuronal development. This mutation alters the secondary structure of mRNA, disrupting a stem loop structure and leading to increased inclusion of exon 10 (15). It will be interesting to investigate whether nonsynonymous and synonymous *MAPT* mutations such as N296H and N296N, which alter tau splicing via disrupting the recruitment of splicing factors, are able to override the developmental regulation of tau splicing in the same way (33). It is also interesting to speculate whether this mutation could lead to a neurodevelopmental cellular phenotype in patients, which precedes disease onset by many years.

Our model will provide an *in vitro* system to understand the functional differences between 3R and 4R tau and the consequences of altered tau splicing. 4R tau isoforms bind microtubules with higher affinity than 3R tau (34,35), so alterations in the 3R:4R ratio will affect the microtubule network and subsequently axonal transport. Indeed, 3R and 4R tau differentially affect mitochondrial transport within the neuron, and increased 4R tau results in increased localization of mitochondria to the cell body and a reduction of mitochondria in the axon (36). It is important to note that many studies have relied on overexpression of tau to investigate its role in axonal transport; however, this can lead to clogging of axons and dramatic reorganization of the neuronal cytoskeleton (37). It is, therefore, crucial to study functional differences between tau isoforms in a system with physiological expression levels of 3R and 4R tau. Further, as correcting aberrant tau splicing could be a possible therapeutic avenue for treatment of 4R tauopathy, our model would provide a drug-screening platform to test novel therapies.

We observed a switch in tau splicing at time points that could be considered postnatal. Although this is consistent with the difference in tau splicing observed in the fetal and postnatal brains, it is not feasible to routinely age cell culture to 1 year for mechanistic and drug discovery purposes. It is, therefore, necessary to devise methods to accelerate the acquisition of mature tau splicing. Several candidate splicing factors that could be involved in the postnatal reprogramming of tau splicing exist, perhaps most promising are the CUG-BP- and ETR-3-like factors and muscleblind-like proteins; both of which are known to act on *MAPT* and have been shown to synergistically coordinate splicing events involved in postnatal heart remodeling (38–40). It is of note that 3D-based cell culture systems have demonstrated increased 4R tau expression when compared with 2D cell cultures (41). It will also be interesting and important to verify if other alternative splicing events associated with postnatal neuronal development occur in our system, such as flip/flop splicing of α-amino-3-hydroxy-5-methyl-4-isoxazolepropionic acid receptor subunits and alternative usage of exon 18 of sodium channel 8A (42,43).

In summary, our study demonstrates the potential of iPSC technology to be useful in modeling FTD caused by mutations in *MAPT,* but also some of the obstacles with respect to developmental regulation of tau expression. Ongoing studies aim to accelerate neuronal differentiation and acquisition of mature tau splice variants in a time frame more suitable to disease modeling; nonetheless, we provide an *in vitro* model suitable for understanding disrupted *MAPT* splicing in FTD.

## Materials and Methods

### Generation of fibroblast lines

Primary fibroblast lines were generated from 4 mm skin punch biopsies, which were obtained under informed consent. Ethical permission for this study was obtained from the National Hospital for Neurology and Neurosurgery and the Institute of Neurology joint research ethics committee (study reference 09/H0716/64). The generation and characterization of fibroblast lines have been previously described (44).

### Reprogramming of fibroblasts into induced pluripotent stem cells

Fibroblasts were reprogrammed into iPSC by viral transduction of cMyc, Oct4, Klf4 and Sox2 as described previously (17). Briefly, fibroblasts were transduced with viral particles and maintained on a mouse embryonic fibroblast feeder layer in hESC medium [KO-DMEM, 20% knockout serum replacement, 2 mm l-glutamine, 1 × nonessential amino acids, 50 μM 2-mercaptoethanol, 50 U ml^−1^ penicillin, 50 μg ml^−1^ streptomycin (all from Invitrogen), and 20 ng ml^−1^ FGF2 (Peprotech)] until colonies with iPSC morphology were observed. Colonies with iPSC morphology were mechanically picked and clonally expanded for further characterization. Karyotyping of iPSC was performed by Cell Guidance Systems, UK. Pluripotent stem cells (PSCs) were subsequently cultured under feeder-free conditions on Geltrex in Essential 8 media (Invitrogen). Cultures were fed daily and passaged every 5–7 days. The hESC line Shef 6 was obtained from the UK Stem Cell Bank, and control iPSCs, also generated using retroviral transduction, were obtained from the laboratory of Dr Tilo Kunath.

### MAPT haplotype analysis

*MAPT* haplotype was determined using a PCR assay to detect the presence of a 238 bp deletion on the H2 background between exons 9 and 10 (18). Genomic DNA was extracted from iPSC using phenol–chloroform extraction. Cells were lysed [110 mm Tris pH 8, 50 mm ethylenediaminetetraacetic acid (EDTA), 100 mm NaCl, 0.5% sodium dodecyl sulfate (SDS) with 0.5 mg/ml of proteinase K (*Qiagen*)] overnight at 37°C for complete protein digestion. DNA was extracted by adding an equal volume of phenol:chloroform:isoamyl alcohol (25:24:1) (#*77617, Sigma*) to lysates followed by precipitation using 100% ethanol and 1/30 of the volume of 3 M sodium acetate. DNA was precipitated for 1 h at −20°C and centrifuged at 13 000*g*_(av)_ for 15 min. DNA was washed three times in 70% ethanol prior to dissolving in TE buffer and stored at 4°C. The PCR was performed with genomic DNA at a final volume of 25 μL under the following conditions: 94°C for 2 min, 35 cycles of 94°C for 1 min, 60°C for 1 min, 72°C for 1 min and a final step of 10 min at 72°C. The concentrations of the forward and reverse primers were at 10 mm, and the sequences for both the primers were as follows: forward 5′ GGAAGACGTTCTCACTGATCTG and reverse 5′ AGGAGTCTGGCTTCAGTCTCTC. All PCRs were performed with GoTaq^®^ Hot Start Colorless Master Mix, and they were run in a 1.5% agarose gel with a 100 bp ladder.

### Differentiation of iPSC into cortical neurons

PSCs were differentiated into cortical neurons using dual SMAD inhibition followed by *in vitro* neurogenesis, as described previously (14,45). Briefly, PSCs were grown to 100% confluence before the media was changed to neural induction media [A 1:1 mixture of N-2 and B-27-containing media supplemented with the SMAD inhibitors dorsomorphin (1 μM) and SB431452 (10 μM Tocris). N-2 medium consists of DMEM/F-12 GlutaMAX, 1 × N-2, 5 μg ml^−1^ insulin, 1 mm l-glutamine, 100 μ nonessential amino acids, 100 M 2-mercaptoethanol, 50 U ml^−1^ penicillin and 50 mg ml^−1^ streptomycin. B-27 medium consists of Neurobasal, 1 × B-27, 200 mm l-glutamine, 50 U ml^−1^ penicillin and 50 mg ml^−1^ streptomycin (all from Invitrogen).] Media was changed daily for a 12-day period during which time PSCs were converted to a neuroepithelial layer. At Day 12, cells were replated onto laminin-coated plates using dispase (Invitrogen), and cells were fed every 2 days with neural maintenance media (A 1:1 mix of N2 and B27, as described). Cells were replated with accutase (Invitrogen) once a substantial amount of neurogenesis has occurred (around Day 28) and then replated for the final time at Day 35 onto poly-ornithine and laminin-coated plates (Sigma).

### Western blotting

Cells were lysed in 10 mm Tris, pH 7.4, 100 mm NaCl, 1 mm EDTA, 1 mm EGTA, 1% Triton X-100, 10% glycerol, 0.1% SDS, 0.5% deoxycholate, plus protease and phosphatase inhibitors (Roche) for 1 h at 4°C. Proteins were separated on SDS-polyacrylamide gel electrophoresis (PAGE) BisTris gels (NuPAGE Novex, 10% or 4–12%, Invitrogen) and subsequently transferred onto nitrocellulose membranes. Membranes were blocked in phosphate-buffered saline containing 3% milk (PBS-M) for 1 h at room temperature (RT). Membranes were incubated in primary antibody in PBS-M overnight at 4°C. Blots were developed with IRDye 800-conjugated goat anti-rabbit (Rockland, Inc.) or IRDye 680-conjugated goat anti-mouse (Molecular Probes, Eugene, OR, USA) and visualized using an Odyssey Infrared Imaging System (Li-Cor Biosciences). For analysis of tau isoforms, samples were dephosphorylated prior to electrophoresis using lambda protein phosphatase as described previously (46) and separated by SDS-PAGE alongside a recombinant tau ladder (Sigma).

### Immunofluorescence

Cells were grown in eight-well chamber slides (Ibidi) for immunofluorescence. Cells were fixed in 4% PFA for 20 min at RT, washed with PBS and blocked in 5% goat serum and 0.1% Triton X-100 in PBS. Cells were incubated in primary antibody overnight at 4°C (Table 1). Cells were then incubated with Alexa Fluor 488 and 568 antibodies (Invitrogen, 1:500) for 1 h at RT, and nuclei were stained using 4′,6-diamidino-2-phenylindole. Images were obtained using a Zeiss LSM microscope.

**Table 1.** Primary antibodies used in this study

Name	Epitope	Source	Dilution	Species
DAKO	Total tau	DAKO	1:10 000 (WB/IF)	Rabbit
PHF1	Tau pS396/S404	Peter Davies	1:1000 (WB) 1:500 (IF)	Mouse
AT270	Tau pT181	Thermo Scientific	1:1000 (WB)	Mouse
AT8	Tau p202/205	Thermo Scientific	1:1000 (WB)	Mouse
Pax6	Pax6	Covance	1:500 (IF)	Rabbit
Otx1/2	Otx1 and Otx2	Millipore	1:500 (IF)	Rabbit
Ki67	Ki67	BD	1:500 (IF)	Mouse
Tbr1	Tbr1	Abcam	1:300 (IF)	Rabbit
Satb2	Satb2	Abcam	1:100 (IF)	Mouse
Tuj1	βIII-tubulin	Covance	1:10 000 (WB) 1:5000 (IF)	Mouse
Actin	Actin	Sigma	1:10 000 (WB)	Mouse

AD, Alzheimer's disease; CBD, corticobasal degeneration; CNS, central nervous system; FTD, frontotemporal dementia; hESC, human embryonic stem cell; iPSC, induced pluripotent stem cell; MAPT, microtubule-associated protein tau; PSP, progressive supranuclear palsy.

### Reverse transcriptase-polymerase chain reaction

To determine the presence of tau isoforms +/− exon 10, semi-quantitative RT-PCR was performed using primers located in exons 9 and 13 of *MAPT*, as described previously (47). Total RNA was extracted from cells using Trizol (Life Technologies) according to the manufacturer's protocol. Reverse transcription was performed with the SuperScript III first strand kit (Invitrogen) with oligo(dT) or an equimolar ratio of oligo(dT) and random hexamers. Each reaction contained 0.5–2 μg of RNA in a total volume of 20 μl. Reverse transcription conditions were as follows: incubation at 65°C for 5 min of RNA, random hexamers and 10 mm dNTPs followed by 1 min on ice. The cDNA synthesis Mix [10X RT Buffer, 25 mM MgCl_2_, 0.1 M DTT, RNaseOUT (40 U), SuperScript III RT (200U)] was then added and cDNA synthesis was carried out for 10 min at 25°C, 50 min at 50°C, and 5 min at 85°C to terminate the reaction. To detect MAPT mRNA +/− exon 10, cDNA was amplified with primers located in exon 9 (5′ GTCAAGTCCAAGATCGGCTC 3′) and exon 13 (5′ TGGTCTGTCTTGGCTTTGGC 3′). Glyceraldehyde 3-phosphate dehydrogenase was amplified using the following primers: forward (5′ CCATGGCACCGTCAAGGCTGA 3′) and reverse (5′ GCCAGTAGAGGCAGGGATGAT 3′). Product amplification was obtained using *Taq* DNA polymerase with standard *Taq* buffer (BioLabs). PCR products were separated on 1% agarose gels and stained with GelRed (Biotium).

## Funding

This work was supported by an NC3R CRACK-IT award sponsored by Eli Lilly and Janssen, Alzheimer's Research UK, CBD Solutions, UCB BioPharma and the NIHR Queen Square Dementia Biomedical Research Unit. Funding to pay the Open Access publication charges for this article was provided by the Medical Research Council via the UCL Open Access Fund.
